# SHOOT GRAVITROPISM 5 mediates the stomatal response to darkness in *Arabidopsis*

**DOI:** 10.5511/plantbiotechnology.23.1122a

**Published:** 2024-03-25

**Authors:** Moeca Arai, Keiko Kigoshi, Kosuke Moriwaki, Kyoko Miyashita, Yoshimi Nakano, Sumire Fujiwara

**Affiliations:** 1 Bioproduction Research Institute, National Institute of Advanced Industrial Science and Technology (AIST), Tsukuba, Ibaraki 305-8566, Japan; 2 Graduate School of Science and Technology, University of Tsukuba, Tsukuba, Ibaraki 305-8572, Japan

**Keywords:** INDETERMINATE DOMAINs, SHOOT GRAVITROPISM 5, stomatal closure, transcription factor, water loss

## Abstract

Stomatal regulation, a multifaceted mechanism enabling plants to adapt to diverse environmental conditions and optimize photosynthesis for survival and growth, is considered crucial in drought stress tolerance research. To further enhance our understanding of stomatal regulation, we investigated the novel transcription factors involved in this process. Our findings reveal that *SHOOT GRAVITROPISM 5* (*SGR5*) is involved in the stomatal response to darkness in *Arabidopsis*. Water loss measurements showed that *SGR5*-overexpressing plants retained more water, whereas *SGR5*-knockout lines exhibited increased water loss compared with the control. Unexpectedly, our analyses indicated that SGR5 was not associated with the abscisic acid signaling pathway, in contrast to its homologous transcription factor, INDETERMINATE DOMAIN 14. Instead, *SGR5*-knockout lines exhibited weakened stomatal closure responses upon transition to darkness. Collectively, our results highlight the regulatory role of SGR5 in mediating stomatal movement in response to darkness.

## Introduction

Global climate change is predicted to worsen the agricultural environment and threaten the stable supply of food resources. Drought, a prominent factor that adversely affects plant viability and food production, has been exacerbated by climate change. Consequently, it is imperative to develop plants that are resilient to such abiotic stresses. Enhancing plant productivity is critical to meet the escalating demands of the global population. Although plants have evolved diverse strategies to counter abiotic stress, a comprehensive understanding of these mechanisms is still lacking. Therefore, elucidating the mechanisms underlying drought stress tolerance is crucial for the development of plants that are adapted to climate change (Raza et al. 2019; Razzaq et al. 2021).

Stomata are small pores in the plant epidermis that regulate gas exchange through photosynthesis, respiration, and transpiration. Each stoma consists of guard cells surrounding the pore. The opening and closing of these guard cells control the size of the stomatal pores. Since more than 90% of water loss in plants is due to transpiration through stomata, the control of stomatal response and/or stomatal density are useful method for optimizing water use efficiency and survival under drought stress conditions (Hasanuzzaman et al. 2023; Peng et al. 2022). Stomatal movement is controlled by various factors, including light, humidity, carbon dioxide levels, and internal signaling processes. Light induces stomatal opening, whereas dark conditions induce stomatal closure. Horrer et al. (2016) reported that, in the model plant *Arabidopsis*, starch in guard cells drives plant growth by controlling stomatal aperture. According to this report, in guard cells, starch accumulates from the morning to the early part of the night, gradually decreases during the latter part of the night by dawn, and degrades rapidly within 30 min of exposure to morning light. This night-time accumulation and morning breakdown of starch, distinct from mesophyll cells, are thought to be the driving force behind stomatal opening and closing. In this starch degradation pathway, β-amylase 1 (BAM1) and α-amylase 3 (AMY3) are key enzymes in the blue light-specific stomatal opening, and their mutants exhibit a reduction in starch content level and consequently reduced stomatal apertures (Horrer et al. 2016). Drought stress promotes stomatal closure via abscisic acid (ABA) signaling. ABA triggers the expression of downstream genes whose promoters contain a highly conserved *cis*-element called the ABA-responsive element. ABA-responsive element binding (AREB) proteins, also known as AREB factors (ABFs), are bZIP transcription factors that play pivotal roles in ABA signaling to induce stomatal closure (Chen et al. 2019; Joo et al. 2021).

INDETERMINATED DOMAIN 14 (IDD14) is one of the transcription factors involved in the ABA-dependent drought tolerance of *Arabidopsis*. IDD14 interacts with AREBs/ABFs and positively regulates drought response genes through the ABA signaling pathway (Liu et al. 2022). On the other hand, IDD16, which shares high homology with IDD14, negatively regulates stomatal initiation but not stomatal movement, and its loss-of-function results in drought tolerance (Qi et al. 2019). Phylogenetic analysis has shown that IDD14, IDD16, and SHOOT GRAVITROPISM 5 (SGR5)/IDD15 form highly similar subgroups that diverge from other IDDs (Colasanti et al. 2006). They cooperatively regulate lateral organ morphogenesis by promoting auxin biosynthesis and transport (Cui et al. 2013). These facts suggest that SGR5 presumably functions in drought tolerance in *Arabidopsis*. SGR5 was originally identified as a regulator of early shoot gravitropism by modulating starch accumulation in amyloplasts (Morita et al. 2006; Tanimoto et al. 2008). However, their role in drought tolerance remains unclear.

In this study, with the aim of contributing to the understanding of drought tolerance mechanisms in plants, we investigated novel transcription factors involved in stomatal regulation, thereby revealing the involvement of SGR5 in water loss regulation in *Arabidopsis* as well as the stomatal response to darkness.

## Materials and methods

### Plant materials and growth conditions

All wild-type (WT) plants used in this study were *Arabidopsis* ecotype Col-0 unless otherwise specified. The *SGR5-KO* lines used in this study were *idd15-5*; SALK_087765 (Cui et al. 2013) and *sgr5-3*; SALK_087766 (Morita et al. 2006) obtained from the Arabidopsis Biological Resource Center (ABRC, Columbus, OH, USA). For water loss measurements, plants were grown on Murashige and Skoog (MS) medium (0.5% sucrose and 0.8% Agar) at 22°C under long day (LD) conditions (16 h light/8 h dark) for 2 weeks. For the stomatal aperture measurement, seeds were sawn on MS medium at 22°C under LD conditions for 10 days, and then transplanted to the mixed soil with equal volumes of Supermix A (Sakata Seed, Yokohama, Japan) and vermiculite (Asahi Industry, Ichinomiya, Japan); then, the plants were grown at 22°C under LD conditions for 2 weeks.

### Plasmid construction and plant transformation

Plasmid construction was performed using plant binary vectors based on the gateway cloning technology (Thermo Fisher Scientific, Waltham, MA, USA). The coding sequence of *SGR5* was cloned into pDONR207 using Gateway BP clonase (Thermo Fisher Scientific). To generate *SGR5-OX*, *SGR5* cloned in pDONR207 was transferred into the binary vector pDEST_35S_3fstop_BCKH using Gateway LR clonase II (Thermo Fisher Scientific), and this construct was then used for transformation into WT plants by the floral-dipping method using *Agrobacterium.* Transgenic lines were selected on MS medium containing 30 mg l^−1^ hygromycin. For the co-immunoprecipitation (co-IP) assay, the coding sequence of *ABF1* or *ABF2* was cloned into pDONR207; then, the *ABF*s and *SGR5* sequences cloned in pDONR207 were transferred to pGWB414 (Nakagawa et al. 2007) using Gateway LR clonase II to overexpress 3×hemagglutinin (HA)-tag-fused proteins at the C-terminus. *SGR5* cloned in pDONR207 was transferred to pDEST_35S_3×FLAG_HSP_GWB5 to overexpress the 3×FLAG-tagged proteins at the C-terminus. The pDEST_35S_3×FLAG_HSP_GWB5 vector was generated by inserting a 3×FLAG fragment into the pDEST_35S_HSP_GWB5 vector (Fujiwara et al. 2014). For the gene silencing suppression used in agroinfiltration, the *p19* sequence cloned in pDONR207 was transferred to pDEST_35S_HSP_GWB5 using the Gateway LR reaction.

### Water loss measurement

All plants used in this experiment were grown on MS medium for two weeks. The aerial parts were excised, placed upside down on a small plastic dish, and weighed immediately at 22–24°C under air humidity at 30–40%. The sample weights were measured at specific time points (0, 10, 20, 30, 40, 60, and 90 min). The water loss rate was calculated based on the initial fresh weight of the sample at 0 min.

### β-glucuronidase (GUS) staining

The 3 kb genomic region upstream of the *SGR5* open reading frame was used as a promoter region for its β-glucuronidase (GUS) activity analysis. The *SGR5* promoter was cloned into the pDONRGm-P4P1R vector (Oshima et al. 2011) using BP clonase and transferred into the R4L1pDEST_GUS_BCKK vector (Oshima et al. 2013) using LR clonase II. This construct was transformed into WT plants using the floral-dipping method with *Agrobacterium.* To detect GUS activity, the tissues were immersed in 90% ice-cold acetone and vacuumed, and then incubated for 60–180 min at −20°C. The tissues were rinsed with 50 mM phosphate buffer (pH 7.0) twice, gradually warmed up from −20°C to 22–25°C, and were incubated in GUS staining solution (1 mM X-Gluc, 0.1% Triton X-100, 1 mM ferricyanide, 1 mM ferrocyanide, and 50 mM sodium phosphate buffer) overnight at 37°C. To stop the reaction, the tissues were rinsed with 50 mM phosphate buffer and then dehydrated with increasing concentrations of ethanol (30–70% of ethanol). To make the plant cells transparent, the plants were immersed in a chloral solution of chloral hydrate/glycerol/water (w : w : w=8 : 1 : 2).

### Stomatal aperture measurement

All plants were grown on MS medium at 22°C under LD conditions for 10 days, transferred to the soil and grown for 2 weeks. The 3rd and 4th leaves were harvested under light condition, and the epidermal cells of the abaxial side of the leaves were peeled with the Scotch® Mending Tape (3M Japan, Tokyo, Japan) with a small hole (Ibata et al. 2013). This experiment was performed as previously described (Rui and Anderson 2016). The epidermal peels were incubated in light solution (containing 50 mM KCl, 0.1 mM CaCl_2_, and 10 mM MES-KOH, at pH 6.15) at 22°C for 2 h in the light condition as the pretreatment. The epidermal peels were then subjected to subsequent treatments. For the ABA treatment, ABA (final concentration 50 µM) was added to the light solution under light conditions, and the samples were incubated for 2.5 h. For the dark treatment, the samples were transferred to dark conditions from light conditions and then incubated for 2.5 h. The epidermal peels were observed and photographed under a microscope (BZ-9000; Keyence, Osaka, Japan). Stomatal width and length were measured using the ImageJ software (National Institutes of Health, ver. 1.54d), and stomatal apertures were calculated by dividing the width by length.

### Co-IP assay

Transient protein co-expression in *Nicotiana benthamiana* and the co-IP assay were performed as described previously (Fujiwara and Mitsuda 2017) with some modifications. Briefly, protein extracts were incubated with anti-HA antibody beads (014-23081, Fujifilm, Tokyo, Japan) at 4°C for one hour with gentle agitation. The proteins retained on the beads were separated using sodium dodecyl sulfate-polyacrylamide gel electrophoresis. The blots were probed with the anti-HA-peroxidase (12013819001, Sigma-Aldrich, Darmstadt, Germany) or the anti-DYKDDDDK-peroxidase (019-22394, Fujifilm) and detected with SuperSignal™ West Pico PLUS Chemiluminescent Substrate (Thermo Fisher Scientific). ChemiDoc XRS Plus (Bio-Rad Laboratories, Hercules, CA, USA) was used to obtain chemiluminescent images.

## Results

### SGR5 is involved in water loss regulation

**Figure 1:**
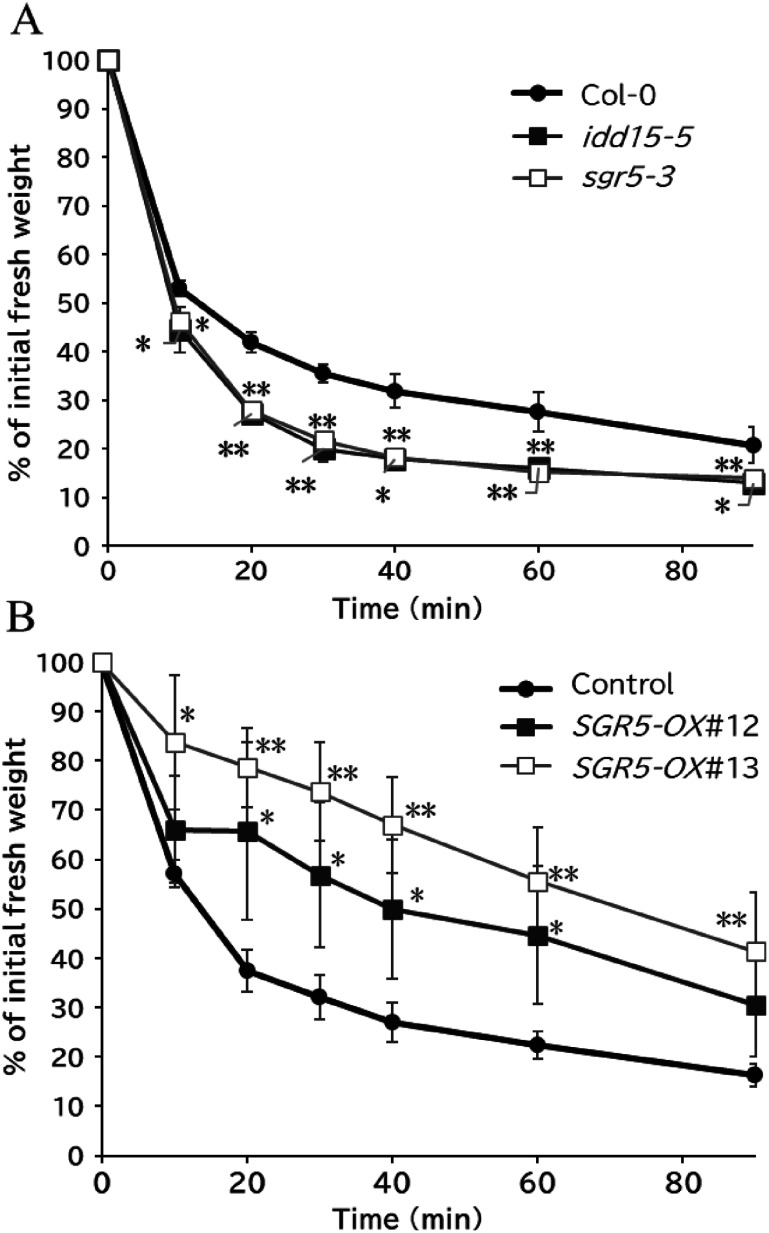
Figure 1. SGR5 plays a positive role in reducing water loss in aerial parts of seedlings. For water loss measurements, the aerial parts of *SGR5-KO* (A) and *SGR5-OX* (B) plants excised from 2-week-old seedlings were weighed at each time point. Water loss was calculated as a percentage of the initial fresh weight. The mean of five replicates is shown, and the bars indicate standard deviation (SD). The experiment was independently repeated three times with similar results. Asterisks indicate significant differences compared to each control line (Welch’s *t*-test, * *p*<0.05, ** *p*<0.01).

In our investigation into the biological functions of C2H2 zinc-finger families, we found that seedlings of loss-of-function SGR5 (*SGR5-KO*) lines, grown on MS plates for two weeks and transferred onto filter paper, exhibited accelerated wilting compared with WT plants. Subsequently, we assessed the water loss rates of the WT and *SGR5-KO* lines. Using the T-DNA insertion lines *idd15-5* (SALK_087765, Cui et al. 2013) and *sgr5-3* (SALK_087766, Morita et al. 2006) (Supplementary Figure S1), we examined the impact of SGR5 loss-of-function. Water loss rate measurements on the detached aerial parts of seedlings revealed that *SGR5-KO* lines had higher rates than WT plants (Figure 1A). To further explore SGR5 function, we generated transgenic *Arabidopsis* plants overexpressing *SGR5* (*SGR5-OX*) and selected lines with the highest *SGR5* expression for further analysis (Supplementary Figure S2A). *SGR5-OX* plants displayed stunted growth (Supplementary Figure S2B, C), with a lower rate of water loss than the vector control line (Figure 1B). These findings suggest that SGR5 plays a positive role in reducing water loss from the aerial parts of the seedlings. Given that over 90% of water loss in plants occurs via stomatal transpiration (Peng et al. 2022), we subsequently explored potential involvement of SGR5 in stomatal movement regulation.

### *SGR5* is expressed in guard cells

**Figure 2:**
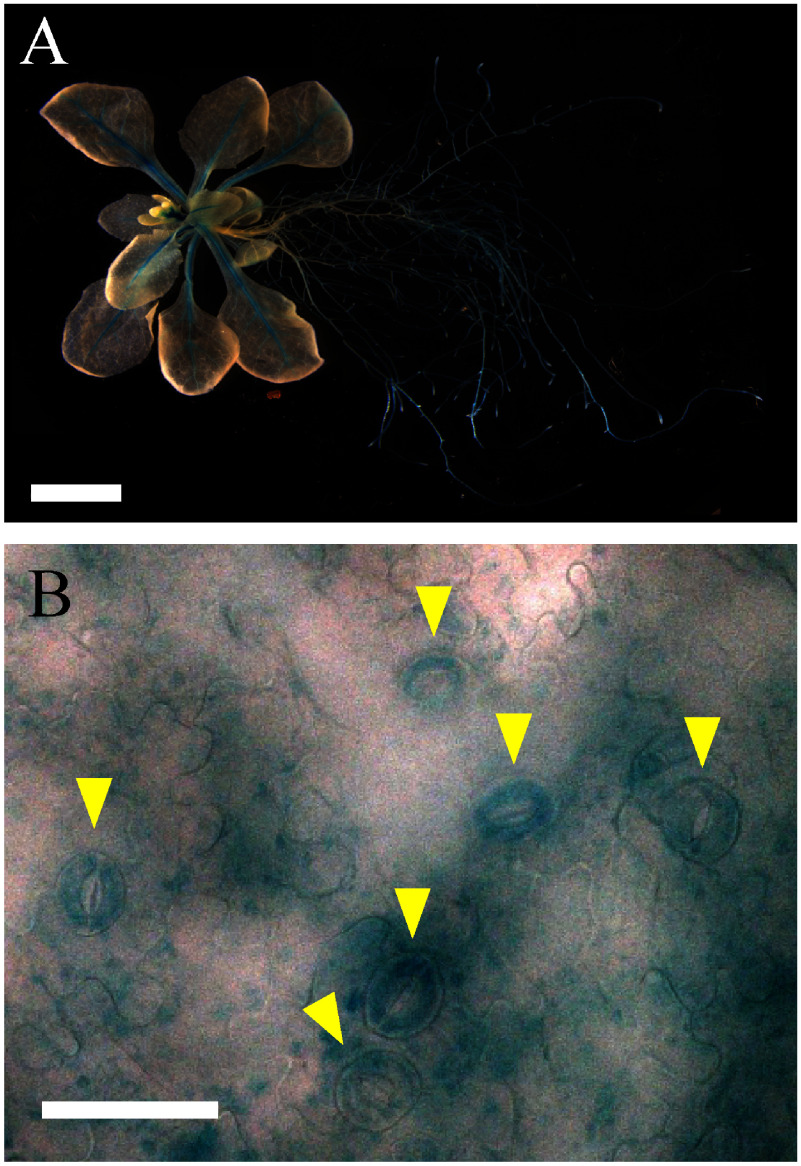
Figure 2. Histochemical localization of *SGR5* promoter:GUS in *Arabidopsis.* Promoter activity of *SGR5* in *SGR5* promoter:GUS transgenic plants (A) and leaves (B). Arrowheads in B indicate the guard cells. Scale bars=5 mm in panel A and 50 mm in panel B.

To further understand the SGR5 function, we investigated the *SGR5* promoter activity in the leaves of *SGR5* promoter:GUS transgenic plants. Strong *SGR5* promoter activity was detected in central leaf veins, consistent with findings from earlier studies (Figure 2A, Cui et al. 2013). Notably, GUS signals were also detected in the guard cells (Figure 2B). Previous transcriptome analyses of guard cells support our results (Adrian et al. 2015; Lee et al. 2019). Therefore, we hypothesized that SGR5 participates in stomatal movement regulation in guard cells.

### SGR5 function may be independent of ABA signaling

**Figure 3:**
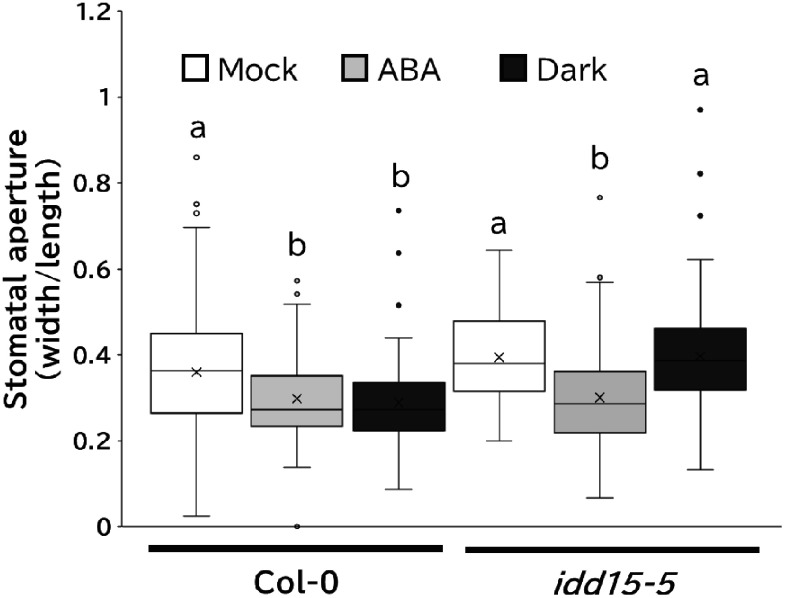
Figure 3. Inhibition of dark-induced stomatal closure through SGR5 loss-of-function. Stomatal aperture measurements of WT plants and *idd15-5* lines. Epidermal peels from the abaxial side of the 3rd and 4th leaves of WT plants and *idd15-5* lines were pretreated under light conditions. Subsequently, plants were treated without (Mock) or with 50 µM ABA (ABA) under light conditions or without ABA under dark conditions (Dark). Following treatment, epidermal peels were observed and photographed under a microscope. The stomatal apertures of 99–212 randomly selected stomata were calculated by dividing the width by the length. Different letters indicate significant differences (pairwise *t*-test with Holm’s correction; *p*<0.05).

To clarify the role of SGR5 in regulating stomatal movement, we examined the effects of ABA on stomatal function in *idd15-5* plants by assessing the stomatal apertures (width/length). ABA is known to induce stomatal closure (Chen et al. 2019; Jezek and Blatt 2017). Consistent with prior studies, our experiments also showed that ABA exposure reduced the stomatal aperture in WT plants (Figure 3). In *idd15-5*, the stomatal aperture under light conditions was similar to that of WT plants, and there were no significant differences in stomatal apertures between *idd15-5* and WT plants following ABA treatment, indicating that ABA-induced stomatal closure occurred in *idd15-5* (Figure 3).

**Figure 4:**
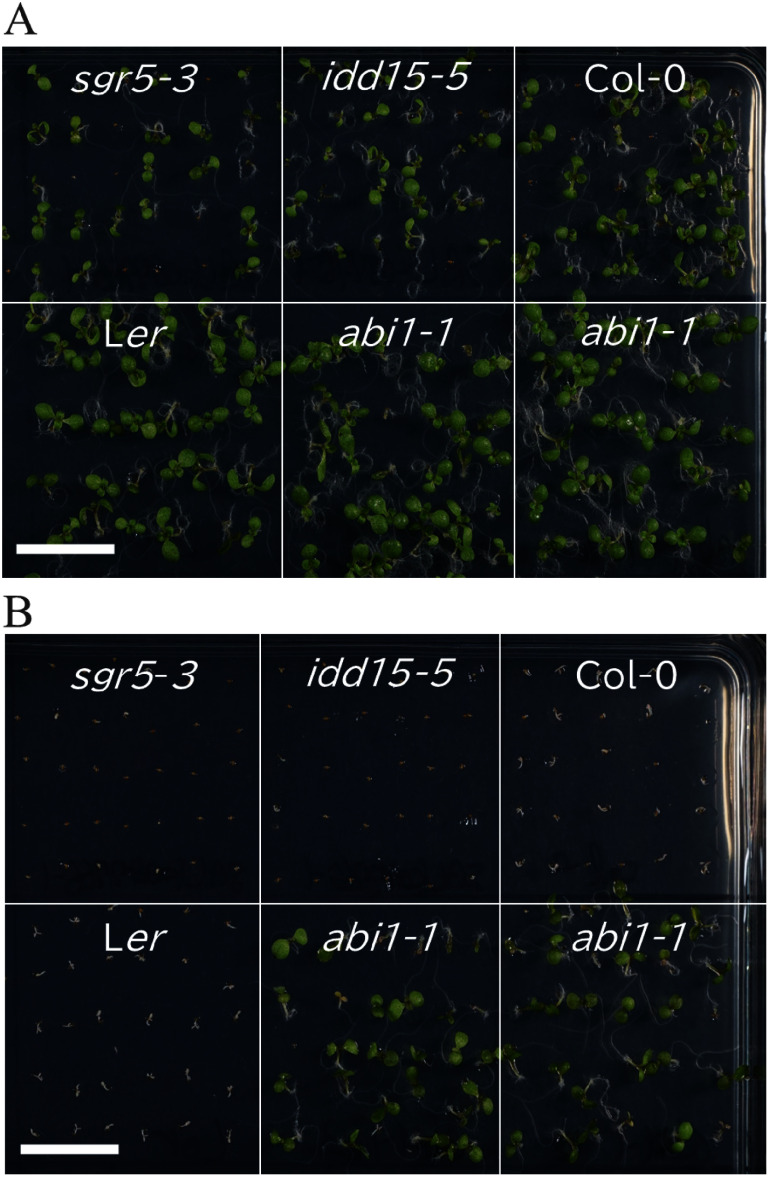
Figure 4. ABA sensitivity of seed germination is unimpaired in *SGR5-KO* lines. Seeds of *SGR5-KO* (*sgr5-3* and *idd15-5*), Col-0 as the control for *SGR5-KO*, *aba insensitive 1* (*abi1*)*-1*, and L*er* as the control for *abi1-1* were grown on half strength MS medium (1% sucrose and 0.8% agar) containing 0 (A) or 5 µM ABA (B). Plates were kept at 4°C under dark conditions for 4 days and subsequently kept at 22°C under LD conditions for 10 days. The *abi1-1* mutant was used as a control for ABA insensitivity. Scale bars: 1 cm.

To further explore the response to exogenous ABA in *SGR5-KO* lines, we tested the effect of ABA treatment on seed germination (Figure 4). We confirmed the germination capability of most seeds on the ABA-free MS medium. However, WT seeds barely germinated on MS medium containing 5 µM ABA, indicating ABA-induced inhibition of seed germination. *abi1-1*, known to exhibit defective ABA signal transduction during germination (Bertauche et al. 1996), germinated on ABA-containing medium. *sgr5-3* and *idd15-5* showed comparable inability to germinate on ABA-containing MS medium. These results indicated that the *SGR5-KO* lines retained the ability to respond to exogenous ABA.

**Figure 5:**
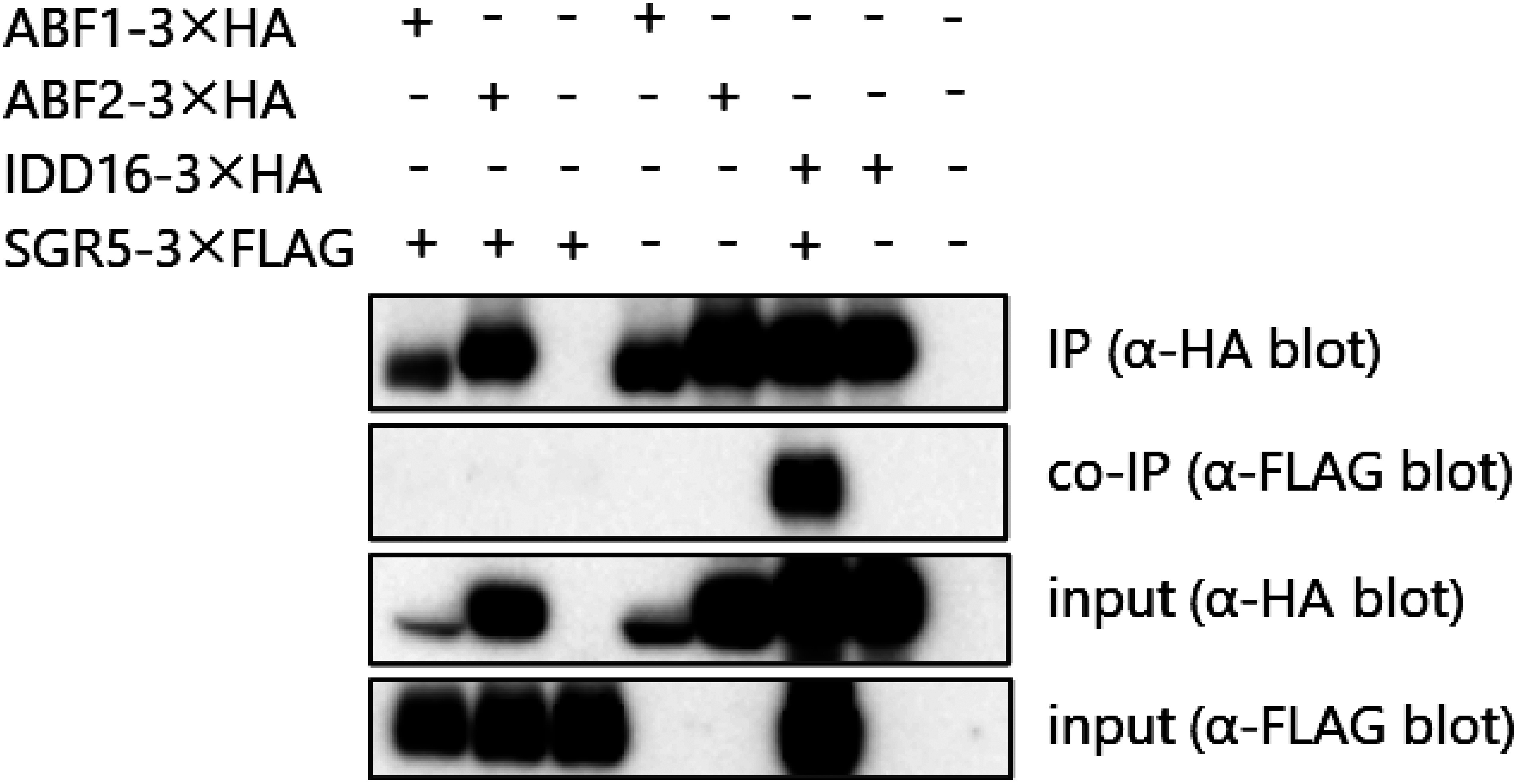
Figure 5. SGR5 does not physically interact with ABFs. Co-IP assays of the interaction between SGR5 and ABF1 or ABF2. Total proteins were extracted from *Nicotiana benthamiana* leaves transiently expressing SGR5-3×FLAG, ABF1-3×HA, or ABF2-3×HA. Protein complexes were co-immunoprecipitated by anti-HA antibody beads and detected by western blot assay with anti-HA or anti-FLAG antibodies. Association between SGR5 and IDD16 (Aoyanagi et al. 2020) was used as a positive control. Representative data from two technical repeats are shown.

Given the lack of significant differences in the effect of ABA treatment between WT and *SGR5-KO* plants, we investigated the physical interaction of SGR5 with ABFs using a co-IP assay, a phenomenon reported for its homolog IDD14, which affects stomatal response through the ABA-mediated pathway (Liu et al. 2022). HA-tagged ABF1, ABF2, and FLAG-tagged SGR5 were coexpressed in *Nicotiana benthamiana* leaves via agroinfiltration. Protein complexes containing HA-tagged proteins were purified using anti-HA antibody beads, and proteins in these complexes were detected using anti-HA and anti-FLAG antibodies. The interaction between SGR5 and IDD16 was also examined, serving as the positive control, given that SGR5 has been shown to physically interact with IDD16 (Aoyanagi et al. 2020). The results revealed the presence of the SGR5–IDD16 physical interaction but a lack of interaction between SGR5 and ABFs (Figure 5), suggesting that SGR5 does not physically interact with ABFs, in contrast to IDD14. Collectively, these findings indicate that SGR5 function may be independent of ABA signaling.

### SGR5 loss-of-function impairs stomatal movement in response to darkness

Not only ABA but also darkness is known to induce stomatal closure (Jezek and Blatt 2017). Notably, significant differences were observed between the WT and *idd15-5* lines when samples were transitioned from light to dark (Figure 3). Stomatal aperture reduction in WT plants was evident, whereas *idd15-5* plants exhibited a significantly larger aperture than WT plants, indicating compromised stomatal closure in response to darkness. This observation implies that SGR5 is involved in the stomatal response to darkness.

## Discussion

In this study, we identified SGR5 as a novel transcription factor that participates in stomatal movement in response to darkness. *SGR5-OX* seedlings retained more water than the control plants (Figure 1B), whereas *SGR5-KO* seedlings lost more water than their WT counterparts (Figure 1A). These findings suggest that SGR5 positively contributes to reducing water loss in the aerial parts of the plants.

Notably, *SGR5-OX* seedlings exhibited stunted growth (Supplementary Figure S2B). Overexpression of IDD14, a close homolog of SGR5, has been reported to induce small and down-curled leaves (Liu et al. 2022), suggesting that ectopic overexpression of *SGR5* and its homolog causes growth suppression. Although dwarfism may lead to reduced transpiration and water consumption, potentially resulting in drought tolerance, further comprehensive experiments are required to validate the effects of *SGR5* overexpression on drought tolerance. Given that *SGR5* is expressed in both aerial parts and roots, it may function in regulating stomatal movements, controlling transpiration, water uptake in roots, and transport from roots. Our investigation primarily focused on *SGR5* function in young seedlings, considering its relatively high expression levels at this stage (Morita et al. 2006).

We observed *SGR5* promoter activity in the guard cells of the seedlings (Figure 2), and previous transcriptome analyses revealed *SGR5* expression in guard cells (Adrian et al. 2015; Lee et al. 2019), indicating its involvement in stomatal control (Figure 2). Subsequent examination of the effects of SGR5 loss-of-function on stomatal movement revealed that the stomatal aperture of *idd15-5* was comparable to that of WT under light conditions, both with and without ABA treatment (Figure 3). A previous study revealed that IDD14 gain of function led to a reduced water loss rate, whereas its loss-of-function resulted in a greater loss of water compared with the WT (Liu et al. 2022). This trend aligns with the observations made in our experiments on SGR5 (Figure 1). These results suggest a functional redundancy between SGR5 and IDD14. However, differences in the mechanisms regulating stomatal movement between the two proteins were apparent. A reduction in ABA sensitivity was observed in *idd14-1* cells, with a higher proportion of stomata in these cells failing to close under ABA treatment (Liu et al. 2022). During periods of drought and osmotic stress, the AREB/ABF family plays a crucial role as transcription factors in the ABA-signaling pathway (Shinozaki and Yamaguchi-Shinozaki 2022). IDD14 physically interacts with AREBs/ABFs, leading to the induction of downstream target gene expression (Liu et al. 2022). Consequently, the heightened water loss observed in the loss-of-function IDD14 mutants can be attributed to the impairment of ABA signaling. In contrast, the loss-of-function of SGR5 did not appear to impair the ABA-signaling pathway, as the responsiveness of *idd15-5* stomata to ABA treatment for closure, along with seed germination of *idd15-5* and *sgr5-3* on ABA-containing media, was comparable to that of WT plants (Figure 4). Furthermore, the co-IP assay revealed no interaction between SGR5 and AREBs/ABFs (Figure 5). Collectively, these results suggest that SGR5 is unlikely to be involved in the ABA-signaling pathway.

Notably, *idd15-5* plants exhibited significantly larger stomatal apertures than WT plants when transferred from light to dark conditions (Figure 3). The stomatal aperture of *idd15-5* plants under dark conditions resembled that under light conditions, indicating an impaired stomatal closure in response to darkness. *SGR5* was originally identified as a transcription factor involved in shoot gravitropism that affects amyloplast sedimentation (Morita et al. 2006). Moreover, *sgr5* mutants accumulate lower amounts of starch than control plants (Tanimoto et al. 2008). Starch metabolism in guard cells, one of the key factors for stomatal movement, involves the activation of H^+^-ATPase and proton efflux under blue light, leading to hyperpolarization of the plasma membrane, K^+^ uptake and accumulation of counterions (Cl^−^, nitrate and malate^2−^) for stomatal opening (Inoue and Kinoshita 2017; Horrer et al. 2016). However, during stomatal closure, these anions discharge through the anion channels. The reduction in malate^2−^ levels is linked to gluconeogenic conversion to starch (Daszkowska-Golec and Szarejko 2013). Starch accumulates at night and rapidly degrades after dawn under blue light exposure, which is a mechanism that controls stomatal opening. Guard cell-specific enzymes, including BAM1 and AMY3, play pivotal roles in starch breakdown in response to light (Horrer et al. 2016). Additionally, inhibition of starch degradation in *bam1* and *amy3* mutants led to a decrease in stomatal aperture and enhanced drought tolerance (Horrer et al. 2016; Prasch and Sonnewald 2015). In *idd15-5* plant guard cells, starch accumulation may be suppressed, particularly under dark conditions, owing to relatively elevated *SGR5* expression levels in the absence of light (Kim et al. 2016). This suppression might have resulted in diminished stomatal response to darkness (Figure 3). During stomatal closure, starch synthesis serves as a sink for osmolytes, including sugars and malate, facilitating the necessary changes in guard cell turgor for water efflux (Santelia et al. 2016). Inhibition of starch accumulation in *idd15-5* plants may impede the proper removal of osmolytes associated with starch synthesis. Although our understanding of the role of starch synthesis and accumulation in stomatal movement has progressed, it remains unclear. Future studies should include an analysis of starch levels in guard cells of *SGR5-KO* plants to further elucidate the intricate interplay between starch dynamics and stomatal regulation.

This study primarily focused on drought tolerance-related stomatal movement. Under light conditions, the *idd15-5* mutant exhibited higher water loss than the WT plants (Figure 1A), despite the stomatal aperture size of *idd15-5* being comparable to that of the WT (Figure 3). Stomatal density may be the key to understanding *idd15-5*’s water loss phenotype under light conditions. In addition to stomatal movement, stomatal density and form also influence drought tolerance. IDD16, another homolog of SGR5, negatively regulates stomatal initiation by regulating the *SPEECHLESS* (*SPCH*) expression, which is crucial for guard cell differentiation (Qi et al. 2019). Although *SGR5* promoter activity was not detected in meristemoid mother cells (Figure 2B), which are regulated by SPCH, previous transcriptome analyses revealed the presence of *SGR5* expression throughout the process of stomatal development and the expression was notably higher than those of *IDD14* and *IDD16* (Adrian et al. 2015; Lee et al. 2019), implying the importance of *SGR5*. Analysis of stomatal density in both *SGR5-OX* and *SGR5-KO* plants is essential, as SGR5 functions redundantly with IDD14 and IDD16 for aerial organ morphogenesis (Cui et al. 2013), and SGR5, IDD14, and IDD16 could form a heterodimer (Figure 5; Aoyanagi et al. 2020). In the present study, we did not investigate the drought tolerance of the single, double, and triple mutants of these homologous IDDs. A comprehensive examination of these mutants may provide insights into the detailed functional diversification and redundancy of SGR5, IDD14, and IDD16 in drought tolerance.

In conclusion, our findings indicate that SGR5 plays a role in dark-induced stomatal closure and functions via a pathway distinct from the ABA-signaling pathway. SGR5 likely contributes to the starch state-dependent stomatal movement, although the transcriptional regulatory mechanisms remain unclear. A detailed analysis of the relationship between SGR5 and starch states in guard cells will provide insights to elucidate this mechanism and aid in the development of drought-tolerant plants.

## Abbreviations

ABA: abscisic acid
ABF: abscisic acid responsive elements binding factor
AMY: α-amylase
AREB: ABA-responsive element binding
BAM: β-amylase
co-IP: co-immunoprecipitation
HA: hemagglutinin
IDD: INDETERMINATE DOMAIN
L*er*: Landsberg *erecta*
LD: long day
PP2AA3: PROTEIN PHOSPHATASE 2A SUBUNIT A3
SGR5: SHOOT GRAVITROPISM 5
SPCH: SPEECHLESS
WT: wild-type

## Description of Supplementary Files

Supplementary Figure S1. Schematic diagram of the *SGR5* gene structure.

Supplementary Figure S2. *SGR5*-overexpressing plants.

Supplementary Table S1. Primer list used in this study.
